# Crystal structures of *fac*-tri­carbonyl­chlorido­(6,6′-dihy­droxy-2,2′-bi­pyridine)­rhenium(I) tetra­hydro­furan monosolvate and *fac*-bromido­tricarbon­yl(6,6′-dihy­droxy-2,2′-bi­pyridine)­manganese(I) tetra­hydro­furan monosolvate

**DOI:** 10.1107/S2056989016011841

**Published:** 2016-07-29

**Authors:** Sheri Lense, Nicholas A. Piro, Scott W. Kassel, Andrew Wildish, Brent Jeffery

**Affiliations:** a University of Wisconsin Oshkosh, Department of Chemistry, 800 Algoma Blvd., Oshkosh, WI 54902, USA; b Villanova University, Department of Chemistry, 800 E. Lancaster Avenue, Villanova, PA 19085, USA

**Keywords:** crystal structure, 6,6′-dihy­droxy-2,2′-bi­pyridine ligand, rhenium complex, manganese complex, hydrogen bonding, selective catalysts for CO_2_ reduction

## Abstract

The structures of two facially coordinated Group VII metal complexes, *fac*-[ReCl(C_10_H_8_N_2_O_2_)(CO)_3_]·C_4_H_8_O (I·THF) and *fac*-[MnBr(C_10_H_8_N_2_O_2_)(CO)_3_]·C_4_H_8_O (II·THF), are reported. In both complexes, the metal ion is coordinated by three carbonyl ligands, a halide ligand, and a 6,6′-dihy­droxy-2,2′-bi­pyridine ligand in a distorted octa­hedral geometry. Both complexes co-crystallize with a non-coordinating tetra­hydro­furan (THF) solvent mol­ecule and exhibit inter­molecular but not intra­molecular hydrogen bonding. In both crystal structures, chains of complexes are formed due to inter­molecular hydrogen bonding between a hy­droxy group from the 6,6′-dihy­droxy-2,2′-bi­pyridine ligand and the halide ligand from a neighboring complex. The THF mol­ecule is hydrogen bonded to the remaining hy­droxy group.

## Abstract 2

The structures of two facially coordinated Group VII metal complexes, *fac*-[ReCl(6,6′-dihy­droxy-2,2′-bi­pyridine)(CO)_3_]·C_4_H_8_O and *fac*-[MnBr(6,6′-dihy­droxy-2,2′-bi­pyridine)(CO)_3_]·C_4_H_8_O, are reported. These complexes are relevant to catalysis for CO_2_ reduction.

## Chemical context   

The *fac*-[Re(α-di­imine)(CO)_3_ *X*]^*n*+^ and *fac*-[Mn(α-di­imine)(CO)_3_ *X*]^*n*+^ (*X* = halide, *n* = 0 or *X* = neutral ligand, *n* = 1) family of complexes are of inter­est as selective catalysts for the reduction of CO_2_ to CO (Bourrez *et al.*, 2011 ▸; Hawecker *et al.*, 1986 ▸; Smieja *et al.*, 2013 ▸; Sampson *et al.*, 2014 ▸; Machan *et al.*, 2014 ▸; Smieja & Kubiak, 2010 ▸). Utilizing substituted α-di­imine ligands in these complexes can optimize complexes sterically or electronically to catalyze the reduction of CO_2_ to CO (Smieja & Kubiak, 2010 ▸; Sampson *et al.*, 2014 ▸) or facilitate formation of supra­molecular assemblies that promote electrocatalytic reduction of CO_2_ (Machan *et al.*, 2014 ▸). The addition of weak Brønsted acids such as water or methanol is necessary for the catalytic turnover of Mn complexes (Smieja *et al.*, 2013 ▸) and significantly increases the catalytic rate of Re complexes (Smieja *et al.*, 2012 ▸). Introducing intra­molecular phenolic groups positioned near the metal atom has been shown to greatly increase the rate at which an iron tetra­phenyl­porphyrin complex catalyzes the reduction of CO_2_ to CO (Costentin *et al.*, 2012 ▸). Recently, the complexes *fac*-[Re(4,4′-dihy­droxy-2,2′-bi­pyridine)(CO)_3_Cl] and *fac*-[Re(6,6′-dihy­droxy-2,2′-bi­pyridine)(CO)_3_Cl] have been synthesized in order to study the effect of proton-responsive ligands in these catalysts and, for the latter complex, the effect of pendant acids positioned near the metal atom (Manbeck *et al.*, 2015 ▸). Unexpectedly, these complexes were found to exhibit reductive deprotonation of the hy­droxy groups. While the crystal structure of *fac*-[Re(4,4′-dihy­droxy-2,2′-bi­pyridine)(CO)_3_Cl] has been reported (Manbeck *et al.*, 2015 ▸), *fac*-[Re(6,6′-dihy­droxy-2,2′-bi­pyridine)(CO)_3_Cl] has not been characterized crystallographically. In this paper we report the synthesis and structural characterization of *fac*-[Re(6,6′-dihy­droxy-2,2′-bi­pyridine)(CO)_3_Cl] as well as the synthesis and structural characterization of the related and previously unknown complex, *fac*-[Mn(6,6′-dihy­droxy-2,2′-bi­pyridine)(CO)_3_Br]. Both complexes co-crystallize with a tetrahydrofuran (THF) solvent molecule.
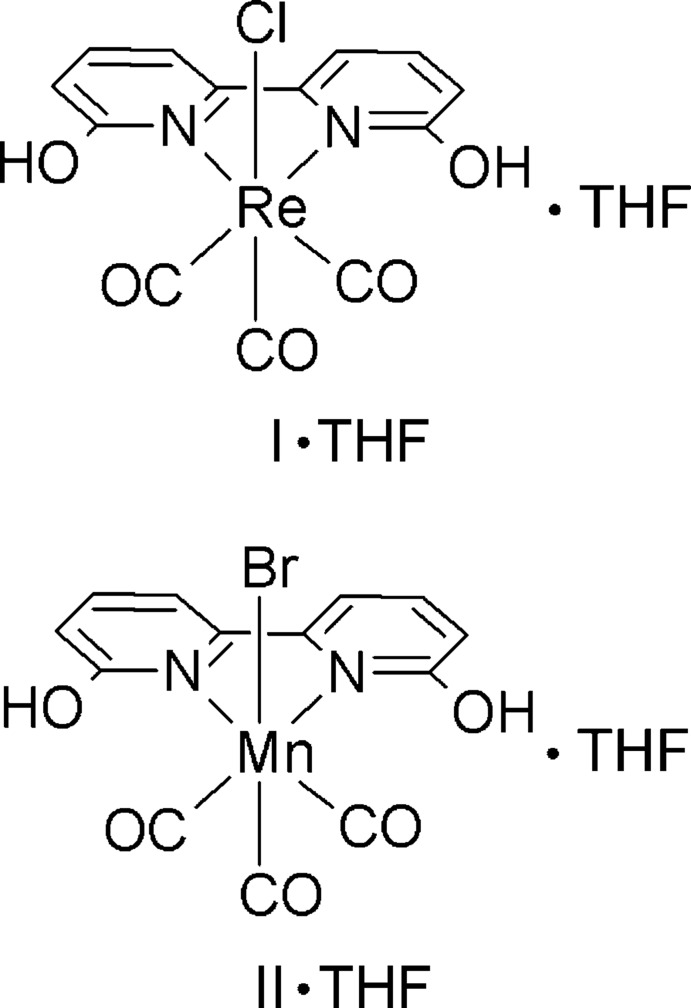


## Structural commentary   

**Figure 1:**
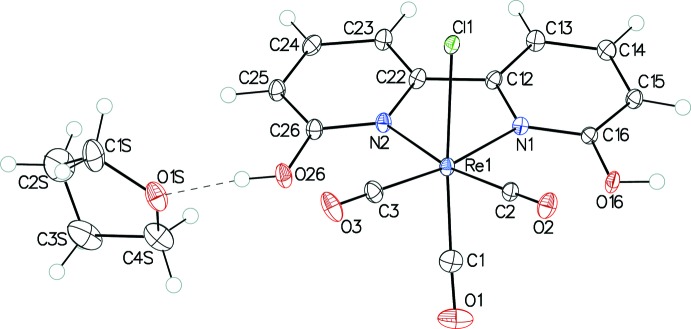
The mol­ecular structure of I·THF, with 50% probability displacement ellipsoids for non-H atoms. The O—H⋯O hydrogen bond is shown by a dashed line. For the THF mol­ecule, only one disordered component is shown.

Figs. 1 ▸ and 2 ▸ show ellipsoid plots of *fac-*[Re(6,6′-dihy­droxy-2,2′-bi­pyridine)(CO)_3_Cl]·THF (I·THF) and [Mn(6,6′-dihy­droxy-2,2′-bi­pyridine)(CO)_3_Br]·THF (II·THF), respectively. Complexes I and II exhibit distorted octa­hedral geometries and contain primary coord­ination spheres similar to those of other *fac*-[Re(α-di­imine)(CO)_3_Cl] and *fac*-[Mn(α-di­imine)(CO)_3_Br] complexes, including [Re(bi­pyridine)(CO)_3_Cl] (III) (Manbeck *et al.*, 2015 ▸), [Re(4,4′-dihy­droxy-2,2′-bi­pyridine)(CO)_3_Cl]·DMSO (IV) (Manbeck *et al.*, 2015 ▸) and [Mn(bi­pyridine)(CO)_3_I] (V) (Stor *et al.*, 1995 ▸). Many coordination modes are possible for the 2-hy­droxy­pyridine ligand (Parsons & Winpenny, 1997 ▸), but the crystal structures confirm bidentate α-di­imine coordination in both complexes. Bond lengths between the metal and bipyridyl nitro­gen atoms are slightly longer in I [2.198 (2) and 2.206 (2) Å] and II [2.0605 (11) and 2.0757 (11) Å] than in complexes III [2.176 (6) and 2.173 (6) Å], IV [2.177 (3) and 2.163 (3) Å] and V [2.05 (1) and 2.03 (2) Å], which do not have substituents in the 6 and 6′ positions on the α-di­imine ligand. The longer bond lengths in I and II may be attributed to increased steric encumbrance due to these substituents. In both I and II, the distances between the oxygen atoms of the hy­droxy substit­uents and the carbon atoms of the carbonyl ligands *cis* to the α-di­imine ligands fall within the sum of the van der Waals radii for carbon and oxygen (Batsanov, 2001 ▸). In I, the O(hy­droxy)—C(carbon­yl) distances are 2.800 (3) and 2.813 (4) Å and in II the O(hy­droxy)—C(carbon­yl) distances are 2.660 (2) and 2.615 (2) Å.

In I, the bi­pyridine rings present a bite angle of 74.09 (8)° to Re, similar to that found in III [74.41 (9)°] and IV [74.9 (2)°]. The bi­pyridine–Mn bite angle in II, 78.35 (4)°, is similar to that in V [79.0 (5)°]. The bi­pyridine ligands are not strictly planar. The dihedral angles between the pyridine rings are 11.68 (9)° in I and 9.49 (5)° in II. Additionally, the bi­pyridine ligands are not oriented strictly perpendicularly to the coordination planes of the metal ions. The dihedral angles between the mean plane through the α-di­imine ligands and the CO_equatorial_–*M*–CO_equatorial_ planes are 23.51 (7) and 18.93 (3)° for the Re and Mn complexes, respectively. Neither I·THF nor II·THF exhibit intra­molecular hydrogen bonding.

## Supra­molecular features   

**Figure 3:**
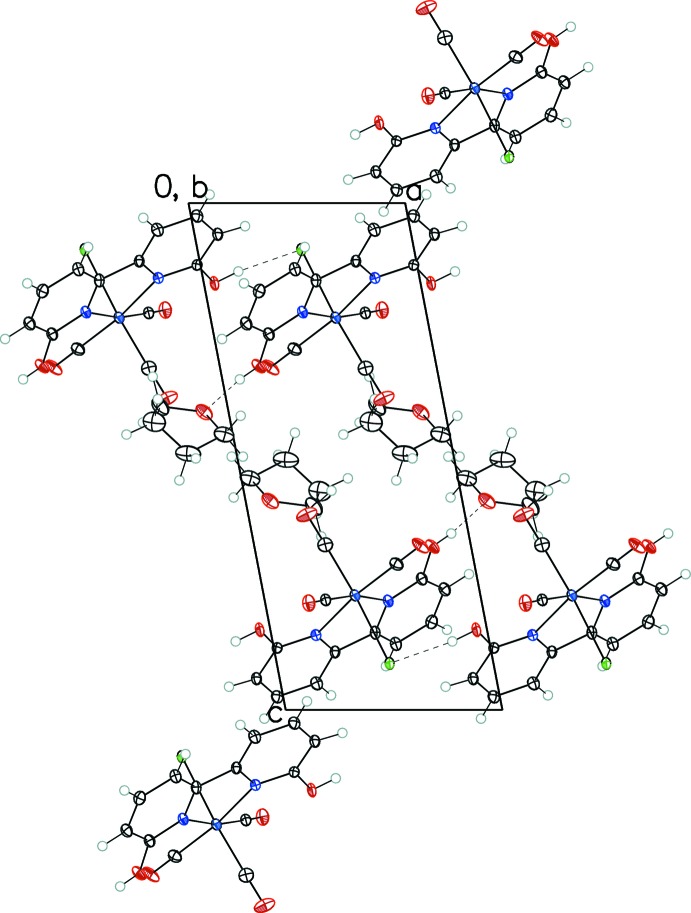
Crystal packing diagram of I·THF viewed along the *b* axis, showing hydrogen bonding (dashed lines) in the structure.

Hydrogen bonds for both structures are listed in Tables 1 ▸ and 2 ▸. In I·THF, a chain of complexes running along the *a* axis is formed by an O—H⋯Cl hydrogen bond between a hy­droxy group (O16—H16) and the chloride ligand from the neighboring complex. The other hy­droxy group (O26—H26) is hydrogen-bonded to the O atom of the THF mol­ecule. The nearest pyridine rings between neighboring complexes have centroid–centroid distances of 3.9448 (16) Å, longer than the maximum distance typically given for π–π inter­actions (Janiak, 2000 ▸). In II·THF, a chain of complexes is formed along the *b* axis through an O—H·Br hydrogen bond involving the O10—H1group and the bromide ligand of the adjacent mol­ecule, whereas the other hy­droxy group (O20—H2) is hydrogen-bonded to O1*S* of the solvent THF mol­ecule. There are weak π–π stacking inter­actions between pairs of complexes from neighboring chains. The centroid–centroid distance between pairs of pyridine rings is 3.7019 (9) Å and the angle between the ring normal and the vector between the ring centroids is 9.3°, within the parameters typically given for such π–π inter­actions (Janiak, 2000 ▸). Packing diagrams are shown in Figs. 3 ▸, 4 ▸ and 5 ▸.

## Synthesis and crystallization   

Methanol was degassed by sparging with N_2_. THF and diethyl ether were dried over mol­ecular sieves and degassed using the freeze–pump–thaw method. MnBr(CO)_5_ and ReCl(CO)_5_ were purchased commercially and used as received. The ligand 6,6′-dihy­droxy-2,2′-bi­pyridine was synthesized according to the synthetic procedure of Umemoto *et al.* (1998 ▸).

I·THF: 6,6′-dihy­droxy-2,2′-bi­pyridine (249 mg, 1.32 mmol) and ReCl(CO)_5_ (477 mg, 1.32 mmol) were heated at 333 K in 50 mL methanol under nitro­gen for five h. The flask was covered with aluminum foil to keep out light. The reaction was then allowed to cool to room temperature and the solvent was removed under vacuum to give a yellow precipitate. Slow cooling of a hot THF solution of the complex in a glove box under a nitro­gen atmosphere gave yellow plate-shaped crystals suitable for single crystal X-ray diffraction. Due to limited solubility of the complex in THF, this method could not be used for a bulk recrystallization of the complex.

II·THF: 6,6′-dihy­droxy-2,2′-bi­pyridine (100 mg, 0.532 mmol) and MnBr(CO)_5_ (146 mg, 0.532 mmol) were heated at 333 K in 24 mL methanol under nitro­gen for five h. The flask was covered with aluminum foil to keep out light. The reaction was then allowed to cool to room temperature and the solvent was removed under vacuum to give an orange precipitate. The complex was recrystallized in bulk by layering pentane on a THF solution of the complex in a glove box under a nitro­gen atmosphere at room temperature, giving the pure product in near qu­anti­tative yield. Slow diffusion of diethyl ether into a THF solution of the complex in a glove box under a nitro­gen atmosphere gave yellow rod-shaped crystals suitable for single crystal X-ray diffraction.

## Refinement   

Crystal data, data collection and structure refinement details are summarized in Table 3 ▸. For both complexes, the coordinates of H atoms forming hydrogen bonds (the hy­droxy group hydrogens) were refined freely with *U*_iso_(H) = 1.5 *U*_eq_(O). C-bound H atoms were placed in calculated positions and refined with riding coordinates, with *U*_iso_(H) = 1.2 *U*_eq_(C). In I·THF, disorder occurs for one carbon and six hydrogens of the THF solvent with occupancies of 0.748 (11) and 0.252 (11). Rigid bond (*DELU*) and similar ADP (*SIMU*) restraints were used for atoms O1*S*, C1*S*, C2*S*, C3*T*, C3*S* and C4*S*.

## Supplementary Material

Crystal structure: contains datablock(s) Re_complex, Mn_complex, global. DOI: 10.1107/S2056989016011841/is5457sup1.cif

Structure factors: contains datablock(s) Re_complex. DOI: 10.1107/S2056989016011841/is5457Re_complexsup4.hkl

Structure factors: contains datablock(s) Mn_complex. DOI: 10.1107/S2056989016011841/is5457Mn_complexsup5.hkl

CCDC references: 1495018, 1495017

Additional supporting information:  crystallographic information; 3D view; checkCIF report

## Figures and Tables

**Figure 2:**
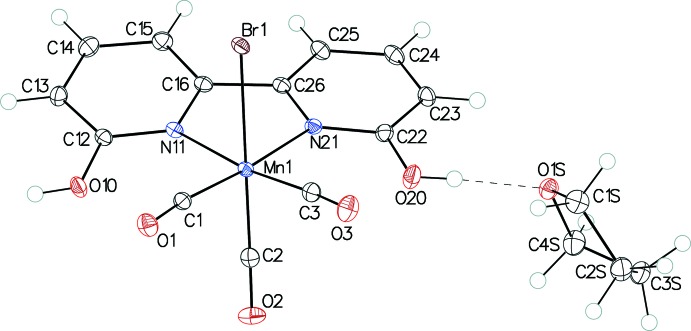
The mol­ecular structure of II·THF, with 50% probability displacement ellipsoids for non-H atoms. The O—H⋯O hydrogen bond is shown by a dashed line.

**Figure 4:**
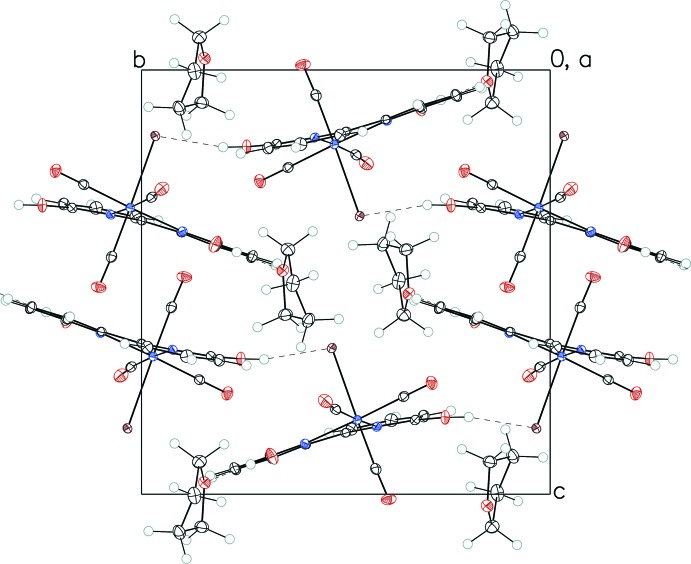
Crystal packing diagram of II·THF viewed along the *a* axis, showing hydrogen bonding (dashed lines) in the structure.

**Figure 5:**
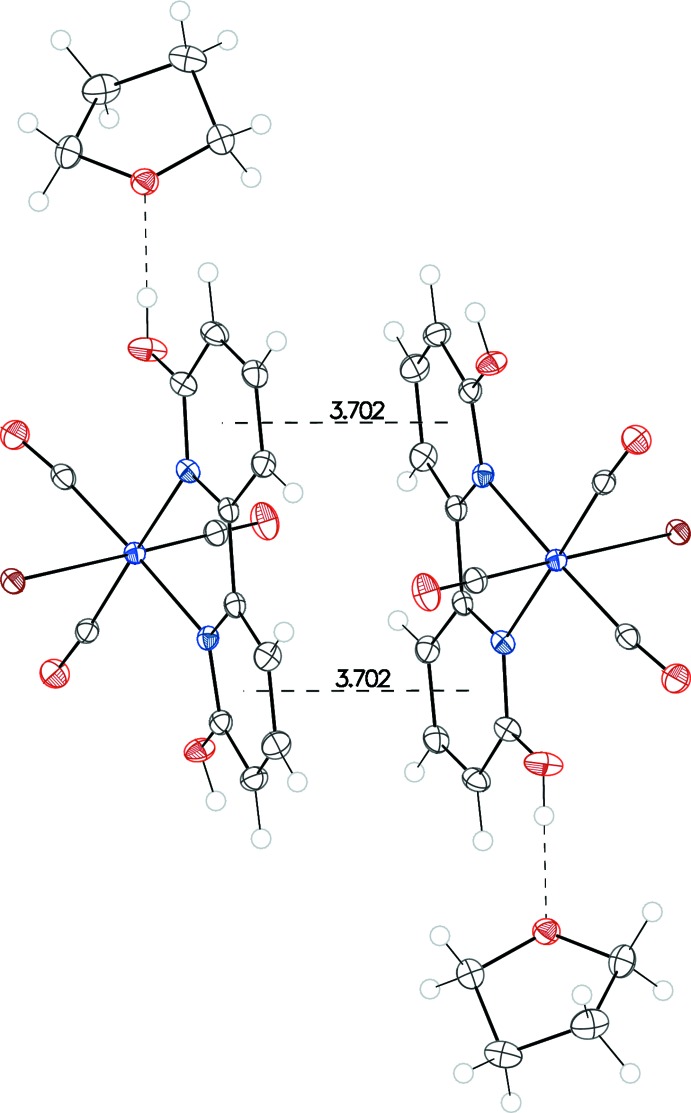
Illustration of π–π stacking inter­actions and O—H⋯O hydrogen bonds (dashed lines) in II·THF.

**Table 1:** Hydrogen-bond geometry (Å, °) for I·THF[Chem scheme1]

*D*—H⋯*A*	*D*—H	H⋯*A*	*D*⋯*A*	*D*—H⋯*A*
O16—H16⋯Cl1^i^	0.86 (4)	2.16 (4)	3.015 (2)	173 (3)
O26—H26⋯O1*S*	0.87 (4)	1.84 (4)	2.704 (3)	173 (4)

Symmetry code: (i) 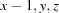.

**Table 2:** Hydrogen-bond geometry (Å, °) for II·THF[Chem scheme1]

*D*—H⋯*A*	*D*—H	H⋯*A*	*D*⋯*A*	*D*—H⋯*A*
O10—H10⋯Br1^i^	0.83 (2)	2.39 (2)	3.2098 (10)	170 (2)
O20—H20⋯O1*S*	0.80 (2)	1.79 (2)	2.5903 (15)	176 (2)

Symmetry code: (i) 
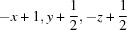
.

**Table 3:** Experimental details

	I·THF	II·THF
Crystal data		
Chemical formula	[ReCl(C_10_H_8_N_2_O_2_)(CO)_3_]·C_4_H_8_O	[MnBr(C_10_H_8_N_2_O_2_)(CO)_3_]·C_4_H_8_O
*M*_r_	565.97	479.17
Crystal system, space group	Triclinic, *P* 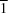	Monoclinic, *P*2_1_/*c*
Temperature (K)	100	100
*a*, *b*, *c* (Å)	6.9661 (6), 8.0082 (6), 16.9007 (13)	10.2401 (12), 13.1783 (15), 14.2480 (16)
α, β, γ (°)	78.907 (2), 79.128 (2), 88.886 (2)	90, 106.228 (3), 90
*V* (Å^3^)	908.46 (13)	1846.1 (4)
*Z*	2	4
Radiation type	Mo *K*α	Mo *K*α
μ (mm^−1^)	6.87	2.92
Crystal size (mm)	0.20 × 0.10 × 0.01	0.36 × 0.13 × 0.08

Data collection		
Diffractometer	Bruker APEXII CCD	Bruker SMART APEX CCD area-detector
Absorption correction	Multi-scan (*SADABS*; Bruker, 2012 ▸)	Multi-scan (*SADABS*; Bruker, 2012 ▸)
*T*_min_, *T*_max_	0.583, 0.747	0.609, 0.747
No. of measured, independent and observed [*I* > 2σ(*I*)] reflections	37165, 8820, 7416	67915, 7363, 5841
*R*_int_	0.060	0.049
(sin θ/λ)_max_ (Å^−1^)	0.833	0.781

Refinement		
*R*[*F* ^2^ > 2σ(*F* ^2^)], *wR*(*F* ^2^), *S*	0.033, 0.063, 1.01	0.027, 0.061, 1.01
No. of reflections	8820	7363
No. of parameters	260	252
No. of restraints	62	0
H-atom treatment	H atoms treated by a mixture of independent and constrained refinement	H atoms treated by a mixture of independent and constrained refinement
Δρ_max_, Δρ_min_ (e Å^−3^)	1.71, −1.88	0.62, −0.45

Computer programs: *APEX2* (Bruker, 2014 ▸), *SAINT* (Bruker, 2013 ▸), *SHELXT* (Sheldrick, 2015*a* ▸), *SHELXL2014* (Sheldrick, 2015*b* ▸) and *OLEX2* (Dolomanov *et al.*, 2009 ▸).

## supplementary crystallographic information

### (Re_complex) fac-Tricarbonylchlorido(6,6'-dihydroxy-2,2'-bipyridine)rhenium(I) tetrahydrofuran monosolvate Crystal data

**Table:**

[ReCl(C_10_H_8_N_2_O_2_)(CO)_3_]·C_4_H_8_O	*Z* = 2
*M**_r_* = 565.97	*F*(000) = 544
Triclinic, *P*1	*D*_x_ = 2.069 Mg m^−^^3^
*a* = 6.9661 (6) Å	Mo *K*α radiation, λ = 0.71073 Å
*b* = 8.0082 (6) Å	Cell parameters from 5671 reflections
*c* = 16.9007 (13) Å	θ = 2.5–32.4°
α = 78.907 (2)°	µ = 6.87 mm^−^^1^
β = 79.128 (2)°	*T* = 100 K
γ = 88.886 (2)°	Plate, yellow
*V* = 908.46 (13) Å^3^	0.2 × 0.1 × 0.01 mm

### (Re_complex) fac-Tricarbonylchlorido(6,6'-dihydroxy-2,2'-bipyridine)rhenium(I) tetrahydrofuran monosolvate Data collection

**Table:**

Bruker APEXII CCD diffractometer	8820 independent reflections
Radiation source: sealed tube	7416 reflections with *I* > 2σ(*I*)
Graphite monochromator	*R*_int_ = 0.060
Detector resolution: 8 pixels mm^-1^	θ_max_ = 36.3°, θ_min_ = 2.5°
ω and φ scans	*h* = −11→11
Absorption correction: multi-scan (SADABS; Bruker, 2012)	*k* = −13→13
*T*_min_ = 0.583, *T*_max_ = 0.747	*l* = −28→27
37165 measured reflections	

### (Re_complex) fac-Tricarbonylchlorido(6,6'-dihydroxy-2,2'-bipyridine)rhenium(I) tetrahydrofuran monosolvate Refinement

**Table:**

Refinement on *F*^2^	Primary atom site location: structure-invariant direct methods
Least-squares matrix: full	Hydrogen site location: mixed
*R*[*F*^2^ > 2σ(*F*^2^)] = 0.033	H atoms treated by a mixture of independent and constrained refinement
*wR*(*F*^2^) = 0.063	*w* = 1/[σ^2^(*F*_o_^2^) + (0.0215*P*)^2^] where *P* = (*F*_o_^2^ + 2*F*_c_^2^)/3
*S* = 1.01	(Δ/σ)_max_ = 0.003
8820 reflections	Δρ_max_ = 1.71 e Å^−^^3^
260 parameters	Δρ_min_ = −1.88 e Å^−^^3^
62 restraints	

### (Re_complex) fac-Tricarbonylchlorido(6,6'-dihydroxy-2,2'-bipyridine)rhenium(I) tetrahydrofuran monosolvate Special details

**Table:**

Geometry. All esds (except the esd in the dihedral angle between two l.s. planes) are estimated using the full covariance matrix. The cell esds are taken into account individually in the estimation of esds in distances, angles and torsion angles; correlations between esds in cell parameters are only used when they are defined by crystal symmetry. An approximate (isotropic) treatment of cell esds is used for estimating esds involving l.s. planes.

### (Re_complex) fac-Tricarbonylchlorido(6,6'-dihydroxy-2,2'-bipyridine)rhenium(I) tetrahydrofuran monosolvate Fractional atomic coordinates and isotropic or equivalent isotropic displacement parameters (Å^2^)

**Table:**

	*x*	*y*	*z*	*U*_iso_*/*U*_eq_	Occ. (<1)
Re1	0.42194 (2)	0.68445 (2)	0.77393 (2)	0.01301 (3)	
Cl1	0.52023 (8)	0.69909 (8)	0.90827 (4)	0.01477 (11)	
O16	−0.0490 (3)	0.6754 (3)	0.84174 (13)	0.0186 (4)	
H16	−0.170 (5)	0.680 (5)	0.865 (2)	0.028*	
O2	0.2010 (3)	1.0152 (3)	0.78896 (14)	0.0241 (5)	
N1	0.2057 (3)	0.4977 (3)	0.85218 (14)	0.0133 (4)	
O1S	1.1132 (3)	0.3754 (3)	0.58779 (15)	0.0292 (5)	
O1	0.2709 (4)	0.7047 (3)	0.61441 (15)	0.0359 (6)	
O26	0.8204 (3)	0.5231 (3)	0.67795 (14)	0.0225 (4)	
H26	0.920 (6)	0.477 (5)	0.652 (2)	0.034*	
N2	0.5697 (3)	0.4372 (3)	0.78476 (14)	0.0144 (4)	
O3	0.7668 (3)	0.9154 (3)	0.67574 (15)	0.0294 (5)	
C3	0.6398 (4)	0.8253 (4)	0.71185 (18)	0.0202 (5)	
C1	0.3292 (5)	0.6881 (4)	0.6741 (2)	0.0228 (6)	
C12	0.2792 (4)	0.3466 (3)	0.88559 (17)	0.0144 (5)	
C24	0.7513 (4)	0.1272 (4)	0.82643 (18)	0.0189 (5)	
H24	0.8148	0.0231	0.8415	0.023*	
C26	0.7446 (4)	0.4026 (3)	0.74160 (17)	0.0163 (5)	
C23	0.5671 (4)	0.1572 (3)	0.86943 (18)	0.0171 (5)	
H23	0.5019	0.0728	0.9132	0.021*	
C16	0.0154 (4)	0.5269 (3)	0.87765 (16)	0.0143 (5)	
C25	0.8405 (4)	0.2491 (4)	0.76204 (18)	0.0184 (5)	
H25	0.9656	0.2298	0.7317	0.022*	
C2	0.2812 (4)	0.8889 (3)	0.78290 (17)	0.0157 (5)	
C22	0.4806 (4)	0.3117 (3)	0.84742 (17)	0.0149 (5)	
C13	0.1667 (4)	0.2300 (3)	0.94723 (18)	0.0178 (5)	
H13	0.2222	0.1274	0.9708	0.021*	
C15	−0.1072 (4)	0.4124 (3)	0.93829 (17)	0.0166 (5)	
H15	−0.2416	0.4359	0.9543	0.020*	
C14	−0.0279 (4)	0.2646 (3)	0.97415 (18)	0.0175 (5)	
H14	−0.1061	0.1868	1.0171	0.021*	
C1S	1.3097 (5)	0.3276 (5)	0.5990 (2)	0.0357 (8)	
H1SA	1.3186	0.3115	0.6577	0.043*	
H1SB	1.4051	0.4173	0.5674	0.043*	
C2S	1.3515 (7)	0.1634 (5)	0.5683 (3)	0.0506 (11)	
H2SA	1.4910	0.1565	0.5431	0.061*	0.748 (11)
H2SB	1.3147	0.0633	0.6130	0.061*	0.748 (11)
H2SC	1.4472	0.1839	0.5160	0.061*	0.252 (11)
H2SD	1.4068	0.0800	0.6090	0.061*	0.252 (11)
C4S	1.0405 (6)	0.2609 (5)	0.5432 (2)	0.0359 (8)	
H4SA	0.9741	0.3242	0.5000	0.043*	0.748 (11)
H4SB	0.9482	0.1754	0.5806	0.043*	0.748 (11)
H4SC	1.0571	0.3126	0.4841	0.043*	0.252 (11)
H4SD	0.9001	0.2342	0.5649	0.043*	0.252 (11)
C3S	1.2233 (10)	0.1777 (7)	0.5060 (4)	0.0479 (17)	0.748 (11)
H3SA	1.1924	0.0640	0.4964	0.057*	0.748 (11)
H3SB	1.2870	0.2487	0.4533	0.057*	0.748 (11)
C3T	1.164 (2)	0.0969 (18)	0.5559 (11)	0.039 (4)	0.252 (11)
H3TA	1.1015	0.0133	0.6046	0.047*	0.252 (11)
H3TB	1.1842	0.0430	0.5070	0.047*	0.252 (11)

### (Re_complex) fac-Tricarbonylchlorido(6,6'-dihydroxy-2,2'-bipyridine)rhenium(I) tetrahydrofuran monosolvate Atomic displacement parameters (Å^2^)

**Table:**

	*U*^11^	*U*^22^	*U*^33^	*U*^12^	*U*^13^	*U*^23^
Re1	0.01156 (4)	0.01031 (5)	0.01605 (5)	0.00098 (3)	−0.00087 (3)	−0.00155 (3)
Cl1	0.0108 (2)	0.0149 (3)	0.0182 (3)	0.00042 (19)	−0.0026 (2)	−0.0021 (2)
O16	0.0089 (8)	0.0184 (10)	0.0256 (11)	0.0010 (7)	−0.0010 (7)	0.0009 (8)
O2	0.0207 (10)	0.0160 (10)	0.0356 (13)	0.0065 (8)	−0.0050 (9)	−0.0062 (9)
N1	0.0126 (9)	0.0123 (10)	0.0151 (10)	0.0001 (7)	−0.0021 (8)	−0.0033 (8)
O1S	0.0317 (12)	0.0209 (11)	0.0313 (13)	−0.0011 (9)	0.0069 (10)	−0.0085 (9)
O1	0.0600 (18)	0.0264 (13)	0.0262 (13)	−0.0014 (11)	−0.0207 (12)	−0.0042 (10)
O26	0.0205 (10)	0.0160 (10)	0.0242 (11)	0.0053 (7)	0.0071 (8)	0.0014 (8)
N2	0.0122 (9)	0.0123 (10)	0.0180 (11)	−0.0003 (7)	−0.0007 (8)	−0.0030 (8)
O3	0.0298 (12)	0.0213 (11)	0.0291 (13)	−0.0128 (9)	0.0121 (10)	−0.0015 (9)
C3	0.0216 (12)	0.0176 (13)	0.0199 (14)	0.0038 (10)	0.0010 (10)	−0.0050 (10)
C1	0.0285 (15)	0.0139 (13)	0.0257 (15)	0.0011 (10)	−0.0056 (12)	−0.0028 (11)
C12	0.0122 (10)	0.0122 (11)	0.0183 (12)	−0.0001 (8)	−0.0033 (9)	−0.0016 (9)
C24	0.0192 (12)	0.0142 (12)	0.0242 (14)	0.0058 (9)	−0.0063 (11)	−0.0041 (10)
C26	0.0145 (11)	0.0140 (12)	0.0197 (13)	0.0007 (9)	−0.0010 (9)	−0.0035 (10)
C23	0.0150 (11)	0.0118 (11)	0.0230 (14)	0.0006 (9)	−0.0016 (10)	−0.0015 (10)
C16	0.0130 (10)	0.0158 (12)	0.0145 (12)	−0.0003 (8)	−0.0029 (9)	−0.0032 (9)
C25	0.0162 (11)	0.0160 (12)	0.0221 (14)	0.0045 (9)	−0.0006 (10)	−0.0046 (10)
C2	0.0129 (10)	0.0158 (12)	0.0177 (12)	0.0002 (9)	−0.0021 (9)	−0.0025 (10)
C22	0.0125 (10)	0.0105 (11)	0.0220 (13)	−0.0012 (8)	−0.0035 (9)	−0.0033 (9)
C13	0.0173 (12)	0.0116 (11)	0.0225 (14)	−0.0013 (9)	−0.0013 (10)	−0.0006 (10)
C15	0.0128 (11)	0.0173 (12)	0.0192 (13)	−0.0022 (9)	−0.0025 (9)	−0.0029 (10)
C14	0.0168 (11)	0.0144 (12)	0.0200 (13)	−0.0041 (9)	−0.0020 (10)	−0.0008 (10)
C1S	0.0332 (17)	0.0256 (17)	0.043 (2)	0.0059 (13)	0.0074 (15)	−0.0073 (15)
C2S	0.067 (3)	0.033 (2)	0.050 (3)	0.021 (2)	−0.004 (2)	−0.0118 (19)
C4S	0.049 (2)	0.0251 (17)	0.0300 (18)	−0.0054 (15)	0.0007 (16)	−0.0050 (14)
C3S	0.083 (4)	0.027 (3)	0.031 (3)	0.016 (3)	−0.001 (3)	−0.010 (2)
C3T	0.054 (7)	0.018 (6)	0.038 (9)	0.002 (5)	0.015 (6)	−0.009 (6)

### (Re_complex) fac-Tricarbonylchlorido(6,6'-dihydroxy-2,2'-bipyridine)rhenium(I) tetrahydrofuran monosolvate Geometric parameters (Å, º)

**Table:**

Re1—Cl1	2.5159 (7)	C16—C15	1.400 (4)
Re1—N1	2.198 (2)	C25—H25	0.9500
Re1—N2	2.206 (2)	C13—H13	0.9500
Re1—C3	1.920 (3)	C13—C14	1.386 (4)
Re1—C1	1.912 (3)	C15—H15	0.9500
Re1—C2	1.908 (3)	C15—C14	1.378 (4)
O16—H16	0.86 (4)	C14—H14	0.9500
O16—C16	1.337 (3)	C1S—H1SA	0.9900
O2—C2	1.158 (3)	C1S—H1SB	0.9900
N1—C12	1.366 (3)	C1S—C2S	1.507 (5)
N1—C16	1.344 (3)	C2S—H2SA	0.9900
O1S—C1S	1.451 (4)	C2S—H2SB	0.9900
O1S—C4S	1.447 (4)	C2S—H2SC	0.9900
O1—C1	1.140 (4)	C2S—H2SD	0.9900
O26—H26	0.87 (4)	C2S—C3S	1.491 (8)
O26—C26	1.332 (3)	C2S—C3T	1.484 (18)
N2—C26	1.350 (3)	C4S—H4SA	0.9900
N2—C22	1.372 (3)	C4S—H4SB	0.9900
O3—C3	1.152 (4)	C4S—H4SC	0.9900
C12—C22	1.475 (3)	C4S—H4SD	0.9900
C12—C13	1.385 (4)	C4S—C3S	1.512 (7)
C24—H24	0.9500	C4S—C3T	1.559 (15)
C24—C23	1.391 (4)	C3S—H3SA	0.9900
C24—C25	1.372 (4)	C3S—H3SB	0.9900
C26—C25	1.403 (4)	C3T—H3TA	0.9900
C23—H23	0.9500	C3T—H3TB	0.9900
C23—C22	1.383 (4)		
			
N1—Re1—Cl1	82.99 (6)	C14—C13—H13	120.4
N1—Re1—N2	74.09 (8)	C16—C15—H15	120.9
N2—Re1—Cl1	85.17 (6)	C14—C15—C16	118.3 (2)
C3—Re1—Cl1	92.47 (9)	C14—C15—H15	120.9
C3—Re1—N1	171.39 (10)	C13—C14—H14	120.2
C3—Re1—N2	98.30 (10)	C15—C14—C13	119.7 (3)
C1—Re1—Cl1	174.77 (9)	C15—C14—H14	120.2
C1—Re1—N1	96.46 (11)	O1S—C1S—H1SA	110.3
C1—Re1—N2	99.71 (11)	O1S—C1S—H1SB	110.3
C1—Re1—C3	88.72 (13)	O1S—C1S—C2S	106.9 (3)
C2—Re1—Cl1	87.58 (8)	H1SA—C1S—H1SB	108.6
C2—Re1—N1	99.67 (10)	C2S—C1S—H1SA	110.3
C2—Re1—N2	170.95 (10)	C2S—C1S—H1SB	110.3
C2—Re1—C3	87.40 (11)	C1S—C2S—H2SA	111.5
C2—Re1—C1	87.38 (12)	C1S—C2S—H2SB	111.5
C16—O16—H16	106 (2)	C1S—C2S—H2SC	110.2
C12—N1—Re1	115.69 (16)	C1S—C2S—H2SD	110.2
C16—N1—Re1	125.85 (17)	H2SA—C2S—H2SB	109.3
C16—N1—C12	117.9 (2)	H2SC—C2S—H2SD	108.5
C4S—O1S—C1S	109.3 (3)	C3S—C2S—C1S	101.4 (3)
C26—O26—H26	105 (3)	C3S—C2S—H2SA	111.5
C26—N2—Re1	126.82 (18)	C3S—C2S—H2SB	111.5
C26—N2—C22	117.6 (2)	C3T—C2S—C1S	107.7 (6)
C22—N2—Re1	115.29 (16)	C3T—C2S—H2SC	110.2
O3—C3—Re1	177.2 (3)	C3T—C2S—H2SD	110.2
O1—C1—Re1	174.1 (3)	O1S—C4S—H4SA	111.1
N1—C12—C22	115.4 (2)	O1S—C4S—H4SB	111.1
N1—C12—C13	121.9 (2)	O1S—C4S—H4SC	110.6
C13—C12—C22	122.6 (2)	O1S—C4S—H4SD	110.6
C23—C24—H24	120.2	O1S—C4S—C3S	103.5 (4)
C25—C24—H24	120.2	O1S—C4S—C3T	105.8 (7)
C25—C24—C23	119.5 (2)	H4SA—C4S—H4SB	109.0
O26—C26—N2	115.8 (2)	H4SC—C4S—H4SD	108.7
O26—C26—C25	121.9 (2)	C3S—C4S—H4SA	111.1
N2—C26—C25	122.3 (2)	C3S—C4S—H4SB	111.1
C24—C23—H23	120.5	C3T—C4S—H4SC	110.6
C22—C23—C24	118.9 (3)	C3T—C4S—H4SD	110.6
C22—C23—H23	120.5	C2S—C3S—C4S	104.5 (4)
O16—C16—N1	115.2 (2)	C2S—C3S—H3SA	110.9
O16—C16—C15	121.9 (2)	C2S—C3S—H3SB	110.9
N1—C16—C15	122.9 (2)	C4S—C3S—H3SA	110.9
C24—C25—C26	119.1 (2)	C4S—C3S—H3SB	110.9
C24—C25—H25	120.4	H3SA—C3S—H3SB	108.9
C26—C25—H25	120.4	C2S—C3T—C4S	102.5 (9)
O2—C2—Re1	177.8 (2)	C2S—C3T—H3TA	111.3
N2—C22—C12	115.6 (2)	C2S—C3T—H3TB	111.3
N2—C22—C23	122.5 (2)	C4S—C3T—H3TA	111.3
C23—C22—C12	121.8 (2)	C4S—C3T—H3TB	111.3
C12—C13—H13	120.4	H3TA—C3T—H3TB	109.2
C12—C13—C14	119.2 (2)		
			
Re1—N1—C12—C22	−16.1 (3)	C12—C13—C14—C15	1.2 (4)
Re1—N1—C12—C13	168.2 (2)	C24—C23—C22—N2	−0.1 (4)
Re1—N1—C16—O16	10.2 (3)	C24—C23—C22—C12	174.8 (3)
Re1—N1—C16—C15	−168.8 (2)	C26—N2—C22—C12	−172.3 (2)
Re1—N2—C26—O26	−10.9 (4)	C26—N2—C22—C23	2.9 (4)
Re1—N2—C26—C25	169.5 (2)	C23—C24—C25—C26	0.8 (4)
Re1—N2—C22—C12	13.6 (3)	C16—N1—C12—C22	172.0 (2)
Re1—N2—C22—C23	−171.2 (2)	C16—N1—C12—C13	−3.7 (4)
O16—C16—C15—C14	−177.9 (3)	C16—C15—C14—C13	−2.7 (4)
N1—C12—C22—N2	1.6 (4)	C25—C24—C23—C22	−1.7 (4)
N1—C12—C22—C23	−173.7 (3)	C22—N2—C26—O26	175.7 (2)
N1—C12—C13—C14	2.1 (4)	C22—N2—C26—C25	−3.9 (4)
N1—C16—C15—C14	1.0 (4)	C22—C12—C13—C14	−173.4 (3)
O1S—C1S—C2S—C3S	27.5 (5)	C13—C12—C22—N2	177.3 (3)
O1S—C1S—C2S—C3T	−11.9 (8)	C13—C12—C22—C23	2.0 (4)
O1S—C4S—C3S—C2S	34.3 (5)	C1S—O1S—C4S—C3S	−16.8 (4)
O1S—C4S—C3T—C2S	−28.0 (10)	C1S—O1S—C4S—C3T	21.7 (7)
O26—C26—C25—C24	−177.5 (3)	C1S—C2S—C3S—C4S	−37.6 (5)
N2—C26—C25—C24	2.2 (4)	C1S—C2S—C3T—C4S	24.0 (11)
C12—N1—C16—O16	−178.8 (2)	C4S—O1S—C1S—C2S	−6.7 (4)
C12—N1—C16—C15	2.2 (4)		

### (Re_complex) fac-Tricarbonylchlorido(6,6'-dihydroxy-2,2'-bipyridine)rhenium(I) tetrahydrofuran monosolvate Hydrogen-bond geometry (Å, º)

**Table:**

*D*—H···*A*	*D*—H	H···*A*	*D*···*A*	*D*—H···*A*
O16—H16···Cl1^i^	0.86 (4)	2.16 (4)	3.015 (2)	173 (3)
O26—H26···O1*S*	0.87 (4)	1.84 (4)	2.704 (3)	173 (4)

Symmetry code: (i) *x*−1, *y*, *z*.

### (Mn_complex) fac-Bromidotricarbonyl(6,6'-dihydroxy-2,2'-bipyridine)manganese(I) tetrahydrofuran monosolvate Crystal data

**Table:**

[MnBr(C_10_H_8_N_2_O_2_)(CO)_3_]·C_4_H_8_O	*F*(000) = 960
*M**_r_* = 479.17	*D*_x_ = 1.724 Mg m^−^^3^
Monoclinic, *P*2_1_/*c*	Mo *K*α radiation, λ = 0.71073 Å
*a* = 10.2401 (12) Å	Cell parameters from 9928 reflections
*b* = 13.1783 (15) Å	θ = 2.7–33.9°
*c* = 14.2480 (16) Å	µ = 2.92 mm^−^^1^
β = 106.228 (3)°	*T* = 100 K
*V* = 1846.1 (4) Å^3^	Rod, yellow
*Z* = 4	0.36 × 0.13 × 0.08 mm

### (Mn_complex) fac-Bromidotricarbonyl(6,6'-dihydroxy-2,2'-bipyridine)manganese(I) tetrahydrofuran monosolvate Data collection

**Table:**

Bruker SMART APEX CCD area-detector diffractometer	7363 independent reflections
Radiation source: sealed tube	5841 reflections with *I* > 2σ(*I*)
Graphite monochromator	*R*_int_ = 0.049
Detector resolution: 8 pixels mm^-1^	θ_max_ = 33.7°, θ_min_ = 2.1°
ω and φ scans	*h* = −15→15
Absorption correction: multi-scan (SADABS; Bruker, 2012)	*k* = −20→20
*T*_min_ = 0.609, *T*_max_ = 0.747	*l* = −22→22
67915 measured reflections	

### (Mn_complex) fac-Bromidotricarbonyl(6,6'-dihydroxy-2,2'-bipyridine)manganese(I) tetrahydrofuran monosolvate Refinement

**Table:**

Refinement on *F*^2^	0 restraints
Least-squares matrix: full	Hydrogen site location: mixed
*R*[*F*^2^ > 2σ(*F*^2^)] = 0.027	H atoms treated by a mixture of independent and constrained refinement
*wR*(*F*^2^) = 0.061	*w* = 1/[σ^2^(*F*_o_^2^) + (0.0253*P*)^2^ + 0.8571*P*] where *P* = (*F*_o_^2^ + 2*F*_c_^2^)/3
*S* = 1.01	(Δ/σ)_max_ = 0.001
7363 reflections	Δρ_max_ = 0.62 e Å^−^^3^
252 parameters	Δρ_min_ = −0.45 e Å^−^^3^

### (Mn_complex) fac-Bromidotricarbonyl(6,6'-dihydroxy-2,2'-bipyridine)manganese(I) tetrahydrofuran monosolvate Special details

**Table:**

Geometry. All esds (except the esd in the dihedral angle between two l.s. planes) are estimated using the full covariance matrix. The cell esds are taken into account individually in the estimation of esds in distances, angles and torsion angles; correlations between esds in cell parameters are only used when they are defined by crystal symmetry. An approximate (isotropic) treatment of cell esds is used for estimating esds involving l.s. planes.

### (Mn_complex) fac-Bromidotricarbonyl(6,6'-dihydroxy-2,2'-bipyridine)manganese(I) tetrahydrofuran monosolvate Fractional atomic coordinates and isotropic or equivalent isotropic displacement parameters (Å^2^)

**Table:**

	*x*	*y*	*z*	*U*_iso_*/*U*_eq_	
Br1	0.43600 (2)	0.46594 (2)	0.34466 (2)	0.01541 (4)	
Mn1	0.30177 (2)	0.52808 (2)	0.17547 (2)	0.01092 (4)	
O1	0.21509 (11)	0.71004 (8)	0.26247 (8)	0.0207 (2)	
C1	0.25680 (14)	0.64158 (10)	0.23009 (10)	0.0145 (2)	
O2	0.13075 (11)	0.60489 (9)	−0.01225 (8)	0.0236 (2)	
C2	0.20034 (14)	0.57322 (10)	0.05953 (10)	0.0153 (2)	
O3	0.05516 (11)	0.44992 (9)	0.21988 (8)	0.0239 (2)	
C3	0.15176 (14)	0.47493 (10)	0.20007 (10)	0.0161 (2)	
O10	0.45262 (10)	0.74298 (7)	0.18113 (8)	0.0179 (2)	
H10	0.487 (2)	0.8003 (18)	0.1820 (17)	0.044 (6)*	
N11	0.48726 (11)	0.57633 (8)	0.15938 (8)	0.01234 (19)	
C12	0.54018 (14)	0.66975 (10)	0.17494 (9)	0.0138 (2)	
C13	0.67743 (14)	0.69023 (11)	0.18420 (11)	0.0174 (3)	
H13	0.7123	0.7572	0.1967	0.021*	
C14	0.76061 (15)	0.61113 (11)	0.17475 (11)	0.0197 (3)	
H14	0.8547	0.6225	0.1826	0.024*	
C15	0.70589 (14)	0.51419 (11)	0.15355 (11)	0.0176 (3)	
H15	0.7614	0.4590	0.1452	0.021*	
C16	0.56946 (14)	0.49991 (10)	0.14487 (9)	0.0132 (2)	
O20	0.16632 (11)	0.31944 (8)	0.08940 (9)	0.0237 (2)	
H20	0.128 (2)	0.2669 (19)	0.0706 (18)	0.047 (7)*	
N21	0.36778 (11)	0.40010 (8)	0.11925 (8)	0.01280 (19)	
C22	0.29616 (14)	0.31508 (10)	0.08977 (10)	0.0153 (2)	
C23	0.35367 (15)	0.22793 (10)	0.06115 (10)	0.0173 (3)	
H23	0.3014	0.1678	0.0436	0.021*	
C24	0.48671 (15)	0.23105 (10)	0.05895 (10)	0.0175 (3)	
H24	0.5278	0.1730	0.0397	0.021*	
C25	0.56093 (15)	0.32024 (10)	0.08525 (10)	0.0161 (2)	
H25	0.6521	0.3246	0.0818	0.019*	
C26	0.49997 (13)	0.40221 (10)	0.11640 (9)	0.0128 (2)	
O1S	0.03314 (10)	0.15416 (8)	0.02750 (8)	0.01819 (19)	
C1S	−0.07218 (16)	0.14179 (12)	0.07676 (12)	0.0229 (3)	
H1SA	−0.0761	0.2020	0.1174	0.027*	
H1SB	−0.0539	0.0811	0.1195	0.027*	
C2S	−0.20485 (16)	0.12945 (12)	−0.00295 (13)	0.0261 (3)	
H2SA	−0.2536	0.1949	−0.0179	0.031*	
H2SB	−0.2648	0.0793	0.0161	0.031*	
C3S	−0.15910 (16)	0.09175 (12)	−0.08984 (12)	0.0255 (3)	
H3SA	−0.1453	0.0173	−0.0871	0.031*	
H3SB	−0.2257	0.1098	−0.1526	0.031*	
C4S	−0.02636 (17)	0.14755 (13)	−0.07673 (11)	0.0245 (3)	
H4SA	0.0341	0.1097	−0.1077	0.029*	
H4SB	−0.0426	0.2161	−0.1061	0.029*	

### (Mn_complex) fac-Bromidotricarbonyl(6,6'-dihydroxy-2,2'-bipyridine)manganese(I) tetrahydrofuran monosolvate Atomic displacement parameters (Å^2^)

**Table:**

	*U*^11^	*U*^22^	*U*^33^	*U*^12^	*U*^13^	*U*^23^
Br1	0.02180 (7)	0.01121 (5)	0.01197 (6)	0.00286 (5)	0.00268 (5)	0.00115 (4)
Mn1	0.01186 (9)	0.00999 (8)	0.01105 (8)	0.00139 (7)	0.00341 (7)	0.00011 (7)
O1	0.0232 (5)	0.0170 (5)	0.0247 (5)	0.0028 (4)	0.0110 (4)	−0.0031 (4)
C1	0.0152 (6)	0.0146 (5)	0.0138 (6)	−0.0007 (5)	0.0044 (5)	0.0015 (4)
O2	0.0215 (5)	0.0314 (6)	0.0158 (5)	0.0088 (4)	0.0020 (4)	0.0028 (4)
C2	0.0154 (6)	0.0152 (6)	0.0170 (6)	0.0015 (5)	0.0075 (5)	−0.0020 (5)
O3	0.0221 (5)	0.0279 (6)	0.0248 (5)	−0.0059 (4)	0.0115 (5)	−0.0049 (4)
C3	0.0187 (6)	0.0156 (6)	0.0143 (6)	0.0009 (5)	0.0050 (5)	−0.0025 (5)
O10	0.0188 (5)	0.0100 (4)	0.0261 (5)	0.0005 (4)	0.0082 (4)	0.0000 (4)
N11	0.0138 (5)	0.0116 (5)	0.0121 (5)	0.0019 (4)	0.0044 (4)	0.0015 (4)
C12	0.0157 (6)	0.0127 (5)	0.0127 (5)	0.0010 (4)	0.0039 (5)	0.0012 (4)
C13	0.0163 (6)	0.0167 (6)	0.0190 (6)	−0.0034 (5)	0.0044 (5)	0.0001 (5)
C14	0.0141 (6)	0.0231 (7)	0.0222 (7)	−0.0012 (5)	0.0055 (5)	−0.0003 (5)
C15	0.0152 (6)	0.0184 (6)	0.0201 (6)	0.0027 (5)	0.0064 (5)	0.0003 (5)
C16	0.0153 (6)	0.0136 (5)	0.0108 (5)	0.0025 (4)	0.0040 (5)	0.0012 (4)
O20	0.0153 (5)	0.0188 (5)	0.0360 (6)	−0.0021 (4)	0.0057 (5)	−0.0110 (5)
N21	0.0134 (5)	0.0122 (4)	0.0120 (5)	0.0021 (4)	0.0022 (4)	−0.0006 (4)
C22	0.0151 (6)	0.0147 (6)	0.0149 (6)	0.0008 (5)	0.0024 (5)	−0.0024 (5)
C23	0.0216 (7)	0.0123 (5)	0.0172 (6)	0.0011 (5)	0.0041 (5)	−0.0028 (5)
C24	0.0238 (7)	0.0130 (6)	0.0172 (6)	0.0048 (5)	0.0081 (5)	−0.0006 (5)
C25	0.0191 (6)	0.0140 (5)	0.0171 (6)	0.0029 (5)	0.0082 (5)	0.0006 (5)
C26	0.0156 (6)	0.0129 (5)	0.0100 (5)	0.0023 (4)	0.0039 (5)	0.0008 (4)
O1S	0.0168 (5)	0.0190 (5)	0.0194 (5)	−0.0015 (4)	0.0060 (4)	−0.0021 (4)
C1S	0.0224 (7)	0.0264 (7)	0.0229 (7)	0.0003 (6)	0.0114 (6)	0.0015 (6)
C2S	0.0174 (7)	0.0230 (7)	0.0388 (9)	0.0006 (6)	0.0095 (7)	−0.0029 (6)
C3S	0.0230 (7)	0.0209 (7)	0.0285 (8)	−0.0017 (6)	0.0008 (6)	−0.0052 (6)
C4S	0.0294 (8)	0.0264 (8)	0.0185 (7)	−0.0050 (6)	0.0078 (6)	−0.0020 (6)

### (Mn_complex) fac-Bromidotricarbonyl(6,6'-dihydroxy-2,2'-bipyridine)manganese(I) tetrahydrofuran monosolvate Geometric parameters (Å, º)

**Table:**

Br1—Mn1	2.5532 (3)	N21—C22	1.3407 (17)
Mn1—C1	1.8043 (14)	N21—C26	1.3658 (17)
Mn1—C2	1.7895 (14)	C22—C23	1.4023 (18)
Mn1—C3	1.8093 (14)	C23—H23	0.9500
Mn1—N11	2.0757 (11)	C23—C24	1.372 (2)
Mn1—N21	2.0605 (11)	C24—H24	0.9500
O1—C1	1.1487 (16)	C24—C25	1.393 (2)
O2—C2	1.1492 (17)	C25—H25	0.9500
O3—C3	1.1503 (17)	C25—C26	1.3815 (18)
O10—H10	0.83 (2)	O1S—C1S	1.4510 (17)
O10—C12	1.3368 (16)	O1S—C4S	1.4416 (18)
N11—C12	1.3384 (17)	C1S—H1SA	0.9900
N11—C16	1.3648 (16)	C1S—H1SB	0.9900
C12—C13	1.4007 (19)	C1S—C2S	1.516 (2)
C13—H13	0.9500	C2S—H2SA	0.9900
C13—C14	1.376 (2)	C2S—H2SB	0.9900
C14—H14	0.9500	C2S—C3S	1.524 (2)
C14—C15	1.394 (2)	C3S—H3SA	0.9900
C15—H15	0.9500	C3S—H3SB	0.9900
C15—C16	1.3806 (19)	C3S—C4S	1.510 (2)
C16—C26	1.4721 (19)	C4S—H4SA	0.9900
O20—H20	0.80 (2)	C4S—H4SB	0.9900
O20—C22	1.3294 (17)		
			
C1—Mn1—Br1	89.64 (4)	O20—C22—N21	115.09 (12)
C1—Mn1—C3	84.52 (6)	O20—C22—C23	122.35 (12)
C1—Mn1—N11	98.36 (5)	N21—C22—C23	122.56 (13)
C1—Mn1—N21	175.75 (5)	C22—C23—H23	120.6
C2—Mn1—Br1	176.95 (4)	C24—C23—C22	118.85 (13)
C2—Mn1—C1	88.30 (6)	C24—C23—H23	120.6
C2—Mn1—C3	90.13 (6)	C23—C24—H24	120.3
C2—Mn1—N11	96.27 (5)	C23—C24—C25	119.32 (12)
C2—Mn1—N21	94.71 (5)	C25—C24—H24	120.3
C3—Mn1—Br1	87.43 (5)	C24—C25—H25	120.5
C3—Mn1—N11	173.04 (5)	C26—C25—C24	118.99 (13)
C3—Mn1—N21	98.45 (5)	C26—C25—H25	120.5
N11—Mn1—Br1	86.26 (3)	N21—C26—C16	114.61 (11)
N21—Mn1—Br1	87.48 (3)	N21—C26—C25	122.29 (12)
N21—Mn1—N11	78.35 (4)	C25—C26—C16	123.05 (12)
O1—C1—Mn1	173.11 (12)	C4S—O1S—C1S	109.52 (11)
O2—C2—Mn1	176.22 (12)	O1S—C1S—H1SA	110.5
O3—C3—Mn1	173.41 (12)	O1S—C1S—H1SB	110.5
C12—O10—H10	111.3 (16)	O1S—C1S—C2S	106.33 (12)
C12—N11—Mn1	127.17 (9)	H1SA—C1S—H1SB	108.7
C12—N11—C16	117.75 (11)	C2S—C1S—H1SA	110.5
C16—N11—Mn1	114.38 (9)	C2S—C1S—H1SB	110.5
O10—C12—N11	115.28 (12)	C1S—C2S—H2SA	111.1
O10—C12—C13	121.90 (12)	C1S—C2S—H2SB	111.1
N11—C12—C13	122.82 (12)	C1S—C2S—C3S	103.23 (12)
C12—C13—H13	120.8	H2SA—C2S—H2SB	109.1
C14—C13—C12	118.49 (13)	C3S—C2S—H2SA	111.1
C14—C13—H13	120.8	C3S—C2S—H2SB	111.1
C13—C14—H14	120.2	C2S—C3S—H3SA	111.3
C13—C14—C15	119.54 (13)	C2S—C3S—H3SB	111.3
C15—C14—H14	120.2	H3SA—C3S—H3SB	109.2
C14—C15—H15	120.7	C4S—C3S—C2S	102.18 (12)
C16—C15—C14	118.67 (13)	C4S—C3S—H3SA	111.3
C16—C15—H15	120.7	C4S—C3S—H3SB	111.3
N11—C16—C15	122.50 (12)	O1S—C4S—C3S	105.28 (12)
N11—C16—C26	114.58 (11)	O1S—C4S—H4SA	110.7
C15—C16—C26	122.89 (12)	O1S—C4S—H4SB	110.7
C22—O20—H20	111.0 (17)	C3S—C4S—H4SA	110.7
C22—N21—Mn1	126.56 (9)	C3S—C4S—H4SB	110.7
C22—N21—C26	117.88 (11)	H4SA—C4S—H4SB	108.8
C26—N21—Mn1	115.50 (8)		
			
Mn1—N11—C12—O10	−15.18 (17)	C15—C16—C26—C25	−7.6 (2)
Mn1—N11—C12—C13	164.82 (10)	C16—N11—C12—O10	175.02 (11)
Mn1—N11—C16—C15	−165.73 (11)	C16—N11—C12—C13	−4.98 (19)
Mn1—N11—C16—C26	16.04 (14)	O20—C22—C23—C24	176.82 (14)
Mn1—N21—C22—O20	6.44 (18)	N21—C22—C23—C24	−3.0 (2)
Mn1—N21—C22—C23	−173.72 (10)	C22—N21—C26—C16	176.89 (11)
Mn1—N21—C26—C16	−5.87 (14)	C22—N21—C26—C25	−0.53 (19)
Mn1—N21—C26—C25	176.72 (10)	C22—C23—C24—C25	0.1 (2)
O10—C12—C13—C14	−178.55 (13)	C23—C24—C25—C26	2.4 (2)
N11—C12—C13—C14	1.4 (2)	C24—C25—C26—C16	−179.43 (12)
N11—C16—C26—N21	−6.80 (16)	C24—C25—C26—N21	−2.2 (2)
N11—C16—C26—C25	170.60 (12)	C26—N21—C22—O20	−176.65 (12)
C12—N11—C16—C15	5.36 (19)	C26—N21—C22—C23	3.18 (19)
C12—N11—C16—C26	−172.87 (11)	O1S—C1S—C2S—C3S	23.09 (16)
C12—C13—C14—C15	1.9 (2)	C1S—O1S—C4S—C3S	−21.18 (16)
C13—C14—C15—C16	−1.5 (2)	C1S—C2S—C3S—C4S	−34.94 (16)
C14—C15—C16—N11	−2.2 (2)	C2S—C3S—C4S—O1S	34.76 (16)
C14—C15—C16—C26	175.91 (13)	C4S—O1S—C1S—C2S	−1.46 (16)
C15—C16—C26—N21	174.98 (12)		

### (Mn_complex) fac-Bromidotricarbonyl(6,6'-dihydroxy-2,2'-bipyridine)manganese(I) tetrahydrofuran monosolvate Hydrogen-bond geometry (Å, º)

**Table:**

*D*—H···*A*	*D*—H	H···*A*	*D*···*A*	*D*—H···*A*
O10—H10···Br1^i^	0.83 (2)	2.39 (2)	3.2098 (10)	170 (2)
O20—H20···O1*S*	0.80 (2)	1.79 (2)	2.5903 (15)	176 (2)

Symmetry code: (i) −*x*+1, *y*+1/2, −*z*+1/2.
